# Application of Computational Biology and Artificial Intelligence in Drug Design

**DOI:** 10.3390/ijms232113568

**Published:** 2022-11-05

**Authors:** Yue Zhang, Mengqi Luo, Peng Wu, Song Wu, Tzong-Yi Lee, Chen Bai

**Affiliations:** 1 School of Life and Health Sciences, School of Medicine, The Chinese University of Hong Kong, Shenzhen 518172, China; 2 School of Chemistry and Materials Science, University of Science and Technology of China, Hefei 230026, China; 3 Warshel Institute for Computational Biology, Shenzhen 518172, China; 4 South China Hospital, Health Science Center, Shenzhen University, Shenzhen 518116, China; 5 School of Biomedical Engineering, Health Science Center, Shenzhen University, Shenzhen 518055, China

**Keywords:** computational biology, computer-aided drug design (CADD), artificial intelligence-aided drug design (AIDD), deep learning

## Abstract

Traditional drug design requires a great amount of research time and developmental expense. Booming computational approaches, including computational biology, computer-aided drug design, and artificial intelligence, have the potential to expedite the efficiency of drug discovery by minimizing the time and financial cost. In recent years, computational approaches are being widely used to improve the efficacy and effectiveness of drug discovery and pipeline, leading to the approval of plenty of new drugs for marketing. The present review emphasizes on the applications of these indispensable computational approaches in aiding target identification, lead discovery, and lead optimization. Some challenges of using these approaches for drug design are also discussed. Moreover, we propose a methodology for integrating various computational techniques into new drug discovery and design.

## 1. Introduction

**Figure 1:**
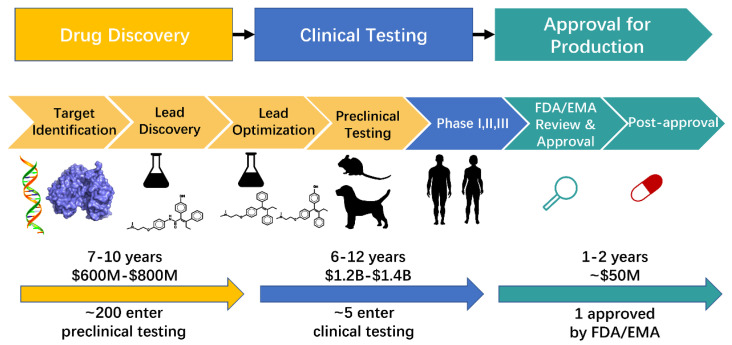
The process of drug research and development. The details in drug development have been improved in the past forty years. Nowadays, the complete process in drug research includes drug discovery, clinical testing, and approval for production. The process of drug discovery includes target identification, lead discovery, lead optimization, and preclinical testing. This process usually takes 7–10 years and $600 M–$800 M. Then approximately 200 compounds enter preclinical testing step while about 5 compounds enter clinical testing process. This process includes three steps, phase I, II and III clinical trials, respectively. It is a long and expensive process that costs 6–12 years and billions of dollars. The compounds that have passed clinical testing enter the process of approval for production. Approved compounds by FDA/EMA can commercialize on the market. This process takes 1–2 years and about $50 M.

Drug research and development is a multistep process that includes drug discovery, clinical testing, and approval for production (Figure 1). Drug discovery is a lengthy, expensive, and complicated process that spans years and costs millions of dollars [1,2]. This process consists of target identification, lead discovery, lead optimization, and preclinical testing (Figure 1) [3,4,5]. Traditional drug discovery begins with the identification of a specific disease, suitable target identification, effective molecule identification (including molecular synthesis and bioactivity testing), and preclinical testing. Despite investing large amounts of money and time, the success rate of clinical testing is below 15% [6]. The cause of failure in approximately 50% of drug discovery is poor pharmacokinetic properties (absorption, distribution, metabolism, excretion, and toxicity [ADMET]) [7]. The speed and success rate of drug discovery have tremendously increased with the development of computational approaches [8].

Nowadays, computational and deep learning approaches play an increasingly vital role in drug discovery. The fast evolvement of methods and algorithms has shortened the time and financial costs in finding the drug candidates.

In drug discovery, contributions of computational biology include the characterization of ligand-binding molecular mechanisms, the identification of binding/active sites and structure refinement of binding poses of the ligand-target. Most of these approaches indicate that binding/active sites on the target protein should be well determined. Specific residues of these binding sites could be used to guide the modification and optimization of the initial lead compound and generate new ligand–target protein interactions. In some cases, engagement of the active site is inadequate for exploring the pathologic activity. Mutations away from the active site, conformational transitions, drug resistance, and expression levels are also known to induce pathosis. Computational biology, especially biomacromolecular simulation, is a powerful method for revealing the molecular mechanism of the target protein and providing new perspectives for drug design.

According to Newtonian Mechanics, molecular dynamics (MD) simulations, which have been widely employed in drug discovery, can capture the position and motion of each atom in a system [9]. This approach can reveal the details of binding, unbinding, and conformational changes of the target protein, which provides complementary information to experiments [10]. Moreover, MD simulations can provide the thermodynamics, kinetics, and free energy profiles of target–ligand interactions [11]. This information can be useful in improving the binding affinity of the lead compound [9]. Due to the availability of more reliable binding affinity results, MD simulations are used to validate the accuracy of docking results [12]. Moreover, quantum mechanics (QM) approaches, such as density functional theory (DFT) [13,14] and ab initio calculation methods [14,15] can be applied to virtual screening (VS) by exploring atomic-electronic interactions between the ligand and target [16]. But these QM approaches are computationally extremely expensive and not always applied to VS in industry. 

Computer-aided drug design (CADD) is typically used to discover, develop, and analyze drug candidates and active molecules having similar biochemical properties [4,17,18,19,20]; accelerate drug discovery, and reduce costs and failure rates [21]. CADD-discovered drug candidates are usually from small-molecule libraries. These discoveries are made using various methods, including molecular docking, pharmacophore modeling, VS, and quantitative structure–activity relationship (QSAR) [4,17,18,19,20]. Among these approaches, VS is the major contributor applied to screen new hit compounds with required properties from large chemical databases. VS is classified as structure-based VS (SBVS) [22,23] and ligand-based VS (LBVS) [24,25]. VS is used to accelerate drug discovery and shorten the number of compounds to be tested in the wet lab. Additionally, VS also plays an important role in drug repurposing or repositioning, optimizing the drug candidates quickly, which accelerates the process of drug design and development [26,27]. 

Artificial intelligence (AI) has recently been proposed as a promising technique in learning and discovering pharmacological big data in drug discovery that has boosted the success rates of drug identification [28]. Using the extensive datasets from biomedical research, AI can learn and discover further rules for translating the data into accessible knowledge. Leading pharmaceutical companies have applied AI to enhance the efficacy of their drug candidates, thereby saving time and costs on unnecessary synthesis and tests. Machine learning [29,30,31], a subfield of AI, and its subfield, deep learning [32,33,34], have been combined with the VS process [35,36,37] to improve the efficiency of similarity searching and the reliability of mining screening data in the ligand-based VS process and enhance the accuracy of scoring functions in structure-based VS [36,37,38]. The approaches also contribute to the generation of novel compounds [32,34].

This review discusses the application of powerful computational approaches to drug discovery and overviews various computational techniques applied to drug discovery including MD simulations, developed Coarse-Grained models, QM, molecular docking, VS, pharmacophore modeling, QSAR, machine learning, and deep learning. Additionally, it discusses how to exploit various computational techniques along with VS to extend the chemical space of novel lead compounds and accelerate the process of drug discovery.

## 2. Computational Biology in Drug Design

Modern drug discovery begins with target identification. Various approaches at the crossroads such as structural biology, molecular biology, cell biology, genomics, proteomics, computational biology, and bioinformatics [39,40,41,42,43,44,45] are being explored to identify the target or related targets and investigate the pathogenesis. Understanding the pathogenesis is also vital for drug discovery and therapies. Computational chemistry techniques, such as MM, QM and MD simulations are widely used in computational biology and medicinal chemistry. MD simulations, DFT, and QM are efficient methods for exploring the pathogenic mechanism and drug resistance [39,46,47,48,49,50,51,52,53,54,55,56,57]. We herein summarize the applications of these methods in drug discovery, specifically in the study of pathogenic mechanism, molecular docking, and lead optimization.

### 2.1. Application of Molecular Mechanics in Drug Design

Molecular mechanics (MM) is an approach which approximately treats the molecules with the laws of classical mechanics and saves the computational resources required for quantum mechanical calculations [58]. Over the past decades, MM approach plays an important role in understanding the ligand-protein structures, interactions and optimizing leads. It is achieved by MM potential energy function, which represents the sum of different energy terms, referred as “force fields” [59]. MM potential energy functions are used in various sampling methods, such as MD and MC (Monte Carlo). MD simulations are often utilized in drug discovery [60,61]. MD is one of the most popular algorithms for sampling. It utilizes various integration algorithms, such as Verlet’s Algorithm, Leap-frog Algorithm and Beeman’s Algorithm, to interpret classical Newton’s equation of motion to analyze the trajectories, movements and interactions in a given molecular system [61]. Time-dependent properties can be obtained from MD [62]. The system is generally a biomacromolecule, such as a protein for example an enzyme, with a solvent environment. For this protein or enzyme system, the initial protein structure is resolved by experiments [63]. Then, the structure could be modelled by different methods. After that, simulations start with the prepared model. X-ray crystallography is used as an experimental method for obtaining the three-dimensional protein structure [64]. However, X-ray requires the protein to form stable crystals and the crystal quality determines the resolution of the structure, which limits the obtainment of high-quality protein structures, especially of membrane proteins [65]. Cryo-EM addresses the problem without the need to form crystals [66,67]. Cryo-EM can determine even quite unstable and intractable membrane protein structures [67]. However, Cryo-EM is not a panacea. In cryo-EM, sample quality is still the most critical factor for determining the high-resolution structure [54]. If no experimental structure is available, modeling or predicting the structure is necessary. Homology modeling [68,69] and AlphaFold developed by DeepMind [70,71,72] are preferred techniques for acquiring the initial protein structure. In the molecular dynamics simulation, atoms and molecules of the system interact during the fixed time, providing the dynamic features of the system. Atom trajectories are generally determined by Newton’s laws of motion. Molecular mechanics methods with various force fields [73,74,75,76,77,78] are employed to calculate the energies of the system.

#### 2.1.1. Application in Investigating the Mechanism of the Target Protein

Target protein can be regulated by drugs to cure the disease or relieve the symptoms. The overall process is dynamic and usually accompanied by the conformational changes of the target protein. Target protein conformation has an essential role in drug design. Even minor changes, as well as the motions of residues, may affect the target–ligand interactions. MD simulations can provide dynamic information about the target protein and the ligand in terms of drug design, which cannot be obtained through experimental methods. Compared with experiments, MD simulations can provide detailed information about the target protein folding process and describe the conformational changes of the protein with environmental changes such as temperature, pH, and residue mutations, with detailed energetic information. At present, MD simulations have been broadly applied to study the molecular mechanisms of the target protein to aid drug design.

For example, Horikoshi et al. revealed the molecular mechanisms underlying the loss of activity in the most severe glucose-6-phosphate dehydrogenase (G6PD) deficiency [79]. It is triggered by the long-distance propagation of structural defects at the dimer interface. The findings indicated that a promising drug can possibly be discovered and developed by correcting these structural defects. While studying pathogenic mutations in the kinesin-3 motor KIF1A by using MD simulations, Budaitis et al. found that these mutations were linked to neurodevelopmental and neurodegenerative disorders that impaired neck linker docking and dramatically reduced the KIF1A force generation [80]. Zanetti-Domingues’s work revealed autoinhibition mechanisms in dimers and oligomers of the epidermal growth factor receptor (EGFR) and supported that dysregulated species bear populations of symmetric and asymmetric kinase dimers coexisting in an equilibrium [81]. The structural feature of the assembly inspires the related drug design. Based on MD simulations, Zhu’s lab elaborated on the genotype-determined EGFR-RTK heterodimerization and its drug resistance mechanism in lung cancer caused by a tighter EGFR-RTK crosstalk [82]. The study promotes drug design against the tighter crosstalk of the genotype determined. Understanding the dynamic behaviors of sirtuins, which have several ligand-binding sites [83], may provide perspectives for the design of selective inhibitors or activators. Polymyxin resistance was found to be induced by lipopolysaccharides and outer membrane vesicles in the multidrug-resistant Pseudomonas aeruginosa [84]. Based on this mechanism, an intelligent strategy for designing lipopeptide antibiotics against polymyxin resistance was developed [84]. The strategy may be suitable for the discovery of other classes of bacterial pathogen-targeting antibiotics. In addition to regular MM approach, coarse-grained models can be used to investigate the molecular mechanism of the target. More details are shown in Section 2.1.4.

#### 2.1.2. Application in Molecular Docking

In molecular docking, according to the space complementarity and energy match, compounds are docked in the specific site. Then, the docking poses are scored and ranked based on the scores [85]. On the basis of molecular docking, VS has been indispensable to drug discovery [86,87]. VS saves time and costs for drug discovery and efficiently obtains various molecule scaffolds [88,89,90,91,92]. The complete VS process consists of pre-processing compound libraries, molecular docking, and the selection of pretest compounds [22,93,94]. In general, the enrichment factor greatly determines the success of VS. The enrichment factor is a validation tool that evaluates the effectiveness of VS by computing the ratio of active molecules among the tested molecules in the initial library. For each VS step, different strategies are used to enhance the enrichment factor [38,95]. The VS results depend on the rationality of the docking poses between the target and ligand, and the accuracy of binding affinity [39,96,97,98,99,100,101,102,103].

After VS step, filtering promising candidates may then be sampled with MD simulations. The use of MD simulations can improve the flexibility in conformational sampling, which increases the number of degrees of freedom of the system and consequently in the computational effort [104]. For MM MD simulations, one of the most time-consuming parts is the calculation of the interactions between each atom in the system, which cost more than 90% of the total simulation time. This is mainly related to the calculation giving rise to O(*N*^2^) computational complexity (*N* represents the number of atoms in the system) [105,106,107]. The cutoff method applied to treat the interactions between atoms can reduce the computational complexity to O(*N*) [105,106]. Compared with the three-dimensional structure of the target protein obtained through X-ray or Cryo-EM, MD simulations take the flexibility of the target protein into account. The experimental structure is in the specific crystalized condition, which is possibly different from the real binding conformation with the ligand. A set of conformations can be obtained by modeling and simulations, especially the crucial intermediate or transition state that may contribute to the ligand–target protein binding process. MD simulations used to sample the specific conformation can provide more rational docking poses and achieve a higher enrichment factor. In addition to the conformation optimization of the target and ligand, MD simulations combined with binding free energy calculations are applied to assess the binding affinity of the ligand with the target. MM-PBSA and MM-GBSA are general approaches used to calculate binding free energy. Based on the trajectories from MD simulations, electrostatic energy, solvation energy, and van der Waals energy are calculated. Entropy change can be obtained through normal mode analysis. Then, the binding free energy can be obtained [108]. The binding free energy calculations are great and useful to augment the accuracy of the binding affinity of docking poses and improve the enrichment factor. But these high-cost sampling calculations are often used on an even smaller subset of potential hits. 

#### 2.1.3. Application in Lead Optimization

The optimal binding mode and the accurate binding affinity are vital for understanding the ligand–target interactions and guiding the modification of screened compounds. The ligand–target thermodynamical data, such as entropy change (ΔS) and free energy change (ΔG), can be determined through experiments and are used to distinguish between active and inactive compounds. However, the lack of details about target–ligand interactions limits further structural modifications of the compounds. MD simulation is a powerful approach for precisely evaluating the ligand–target binding modes. It can describe the detailed ligand–target interactions and determine the free energy contribution of each residue in the binding sites. The information can provide guidance for lead optimization. 

Using the combination of MD simulations and VS, Patel’s lab optimized bedaquiline to decrease the severity of its adverse side effects and discovered that the compound CID 15947587 with low cardiotoxicity has the highest binding free energy (−85.27 kcal/mol) and an optimal docking score (−5.64) with mycobacterial ATP synthase enzyme [109]. Castillo’s group optimized AKT inhibitors by using MD simulations, thereby improving the binding affinity of the 2,4,6-trisubstituted pyridine scaffold in the ATP pocket of PKB/AKT and interacting well with glutamates/aspartates in ATP-binding sites [110]. Zhang et al. screened the new inhibitor against phosphodiesterase-2A (PDE2A). With the guidance of MD simulations, they obtained the optimized lead, LHB-8, forming an extra hydrogen bond with D808 and a hydrophobic interaction with T768, in addition to the interactions with Q859 and F862 of the PDE2A target [111].

#### 2.1.4. Application of Coarse-Grained Models in Drug Design

All-atom MD simulations present the limitation while exploring the dynamic process of the large-scale target protein or long-time scale. Coarse-grained (CG) models help overcome the limitation well. When using CG models, the main chain of residues is in the all-atom state, but the side chain is a simplified united atom. Compared with all-atom MD simulations, CG simulations decrease the number of particles and make the potential energy surface smoother. Thus, the longer time and larger scale are available using CG models. Martini is a classical force filed to employ CG simulations. Martini is currently applied to study the mechanism and oligomerization of membrane proteins and self-assembly of proteins, predict conformational changes, and study binding and pore formation in membranes [112,113,114].

The CG model consistently developed by Warshel et al. [77,78,115] is advantageous in investigating the molecular mechanism of different biophysical systems, such as SARS-CoV-2 [116], GPCR [117], ATPase [118,119], and kinase [120]. This model can accurately describe the electrostatic term [121], which usually is the major contributor compared with other types of interactions in proteins. The CG profile can determine the dynamic information of the reaction in proteins, including the reaction energy barrier, rate-determining step, and the transition state. These results offer energetic details for understanding the working mechanism of proteins and guide rational drug discovery and development. 

We are currently attempting to apply the CG model to VS to obtain more effective compounds. CG simulations can provide vital dynamic information and details about the transition state and energy barrier. The transition state is induced in the molecular docking of VS, and the energy barrier is considered a rule in the score and rank. Then, the selected compounds are used to construct the training set of AI to generate new molecules.

### 2.2. Application of QM in Drug Design

Structural studies have shown that the details of the potential drug target are valuable not only for lead discovery and optimization but also for the later steps of drug design, such as toxicity determination and prediction of binding modes of the leads and drug targets. During drug discovery, the molecular docking or pharmacophore model is used for predicting the binding modes in a short time. MD simulations can be employed to obtain flexible and rational docking modes. They can also guide drug design by exploring ligand–target interactions, such as studying the active site for extra electrostatic, hydrophilic, or hydrophobic interactions that can increase binding affinity [39,122,123]. Although MD simulations improve the accuracy of scoring and docking [124,125,126], concerns still exist, especially in enzymes or metal-containing drug targets, in which valence electron transfer occurs [127,128].

QM is considered the potential solution for the aforementioned concerns, which can explore drug target details at the electronic level [52,123,128]. At present, QM is increasingly applied to enzymes or metal-containing proteins that are considered drug targets, such as HIV-1 protease [129], human DHFR [130], and GPCR [131], and clarify the molecular mechanism for drug design [132,133,134,135]. QM is also used for designing novel drugs, including the high-affinity ligands of FKBP12 [136] and novel inhibitors of human DHFR [137]. 

Additionally, researchers have attempted to improve scoring by inducing QM approaches, especially QM-polarized ligand docking [138], and QMScore, a semiempirical QM (SQM) scoring function [139]. QM in combination with molecular mechanics (MM) has been developed to enhance the accuracy of docking and VS [128,140,141,142]. Fong et al. applied a series of QM/MM scoring functions to six HIV-1 proteases and found that parts of QM/MM functions were superior to MM functions [143]. Kim et al. [144] proposed a new strategy of using QM/MM with the implicit solvation model to rescore docking poses and improve the docking performance on 40 GPCR–ligand complexes. A significant improvement was observed over the conventional docking method. Chaskar et al. developed a QM/MM on-the-fly docking method to deal with polarization and metal interactions in docking and observed a significant improvement in scoring [145]. Compared to MD simulations, QM calculations are even more expensive. For example, the Hartree-Fock recovers approximately 99% of the total electronic energy and requires diagonalizing the *M* by *M* Fock matrix at O(*M*^3^) cost (*M* represents the number of basis functions) [146]. By Shor’s factoring algorithm, the complexity of quantum calculations is O((log_2_*N*)^3^) (*N* represents the number of atoms in the system) [147]. Moreover, QM calculations are restricted to systems of up to a few hundred atoms in contrast to MD simulations, which has evolved from simulating tens of thousands of atoms to handling over 100 million atoms comprising an entire cell organelle [148,149].

## 3. Computer-Aided Drug Design

**Figure 2:**
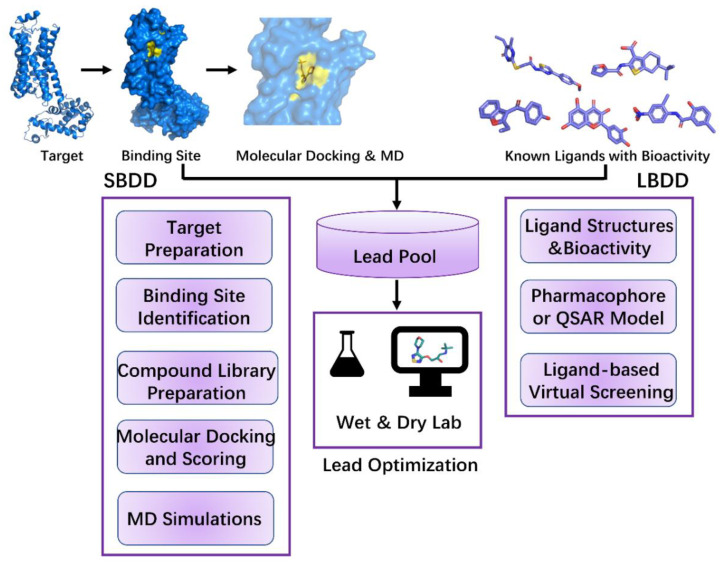
The workflow of structure-based drug design (SBDD) and ligand-based drug design (LBDD). For SBDD, it starts with target identification. Then, binding site of the target requires identifying and compound library needs to be prepared. Next, dock each compound from the library into the identified binding site evaluate the score. In molecular docking, MD simulations can be utilized to obtain more flexible target and rescore for the docking process. Additionally, MD simulations can be applied to lead optimization through ligand-target interactions. Through these steps, leads are obtained primarily. For LBDD, it starts with known ligands with bioactivity. Then, extracting the chemical features of these ligands and build pharmacophore or QSAR model. Next, according to the information of known ligands (e.g., ligand similarity), ligand-based virtual screening is performed in the compound library and leads are screened. These leads are further optimized in wet and dry lab.

CADD has until now led to the discovery of more than 70 approved drugs [4], from Captopril in 1981 [150,151] to Remdesivir in 2021 [152]. Two important categories of CADD, structure-based drug design (SBDD) and ligand-based drug design (LBDD), are highlighted in this review. These two categories have been widely used in lead discovery during drug discovery (Figure 2). SBDD depends on the three-dimensional structure of the target and active sites to determine ligand–target interactions [153]. On the other hand, LBDD is used when the three-dimensional structure of the target is unknown. It begins with a single molecule or a set of molecules effective against the target and depends on the structure–activity relationship [153].

### 3.1. Structure-Based Drug Design

SBDD is an efficient approach for lead discovery and optimization. The most frequently used methods in SBDD, that is, MD simulations, molecular docking, and structure-based VS, are applied to evaluate binding affinity and ligand–target interactions and explore conformational changes in the target. Using SBDD, some approved drugs, such as Imatinib (an abltyrosine kinase inhibitor) [154], Indinavir (Crixivan, the inhibitor of HIV-1 protease) [155], Nilotinib (Tasigna, a selective tyrosine kinase receptor inhibitor used in the treatment of chronic myelogenous leukemia) [156], and Lifitegrast (the LFA-1 antagonist that blocks binding of ICAM-1 to LFA-1) [157], were discovered. SBDD mainly includes target preparation, binding site identification, compound library preparation, molecular docking and scoring, and MD simulations (Figure 2).

#### 3.1.1. Target Preparation

With advancements in structural biology, increasing structures of the target proteins are available and deposited in the PDB. Because of the limitations of experimental approaches, some target structures have not been obtained yet [5,158,159]. Computational approaches such as homology modeling, AlphaFold, and ab initio protein structure prediction can predict target structures according to their sequences [68,69,71,72,160]. Homology modeling selects an appropriate template structure to construct the target structure. AlphaFold is an AI technique developed by DeepMind that predicts three-dimensional protein structures according to their amino acid sequences, which can achieve approximate accuracy as experiments [72]. The ab initio protein structure prediction is considered suitable when the template structure is unavailable in the PDB. This technique considers global optimization, which can help find the tertiary structure with minimum energy based on the primary structure of the specific target [160].

#### 3.1.2. Binding Site Identification

Binding site determination is an essential prerequisite for performing molecular docking. Information about the binding sites of target proteins can be obtained from site-directed mutation and the co-crystallized complex structures of proteins with ligands [161]. When any prior knowledge of the binding pocket is unknown, blind blocking is required to predict the binding sites [162,163,164]. For blind docking method, docking has to be performed on the entire protein surface to find the most probable binding mode. The whole process includes several trials (>100 times recommended by Hetenyi, and Van Der Spoel [165]) and energy evaluations (at least 10 million times per trial recommended by Hetenyi, and Van Der Spoel [165]) to obtain the favorable ligand-target complex pose [163,164,165]. Compared with regular docking, although blind docking is less reliable and limited due to inadequate sampling at the docking space, blind docking is meaningful to discover unexpected interactions that may exist in the unidentified binding modes [164,166,167]. Some tools are developed to predict the binding sites of target proteins by blind docking, including DeepSite [168], DoGSiteScorer [169], POCASA [170], Fpocket [171,172], RaptorX-Binding Site [173], COACH [174], and PocketDepth [175]. 

#### 3.1.3. Compound Library Preparation

Compounds used for VS are selected from compound libraries such as the REAL library of Enamine (1.4 billion make-on-demand compounds) [176], ZINC (750 million purchasable compounds in ready-to-dock) [89,177,178], MCULE (122 million synthetic compounds) [179], PubChem (112 million bioactive compounds) [180,181], DrugBank (14,528 approved drug molecules or experimental drugs) [182,183], ChEMBL (approximately 2.2 million bioactive molecules with drug-like properties) [184,185], and ChemDB (approximately 5 million commercially available small molecules) [186]. The compounds were filtered on the basis of Lipinski’s “Rule of Five” [187], Veber criteria [188], ADMET, and other specific properties (such as carcinogenicity and hepatotoxicity) [158]. Lipinski’s “Rule of Five” and Veber criteria indicate that the compound can be recognized to be orally bioactive if its molecular weight (MW) is <500 Da, hydrogen bond donors (HBD) ≤ 5, hydrogen bond acceptors (HBA) ≤ 10, octanol–water partition coefficient logP ≤ 5, rotatable bonds (RotB) ≤ 10, and topological polar surface area ≤ 140 [187,188]. Moreover, the synthetic accessibility of the compounds should be considered. After filtering ligands from libraries, the optimized 3D structure of the ligand should be modelled.

#### 3.1.4. Molecular Docking and Scoring

Molecular docking is currently used in combination with VS to simplify the search process in the presence of a three-dimensional target structure [87]. It is used to assess ligand–target interactions at the molecular level and rank the ligands according to their binding affinity by using scoring functions [189]. The most frequently used molecular docking tools include Autodock [190], AutoDock Vina [191], CDOCKER [192], GLIDE [193], DOCK6 [194], GOLD [195], FLEXX [196], and SwissDock [197]. Regarding the flexibility of the ligand and target, molecular docking approaches include (1) rigid docking wherein the structures of the ligand and target are both rigid; (2) semi-flexible docking, which is the most commonly used approach, wherein the ligand structure is flexible and the target is rigid; and (3) flexible docking wherein the ligand and target structures are both flexible [198]. Different search algorithms are applied to deal with flexible ligands, such as systematic search algorithms, random or stochastic algorithms, and simulation algorithms [199]. To treat the flexible protein, molecular dynamic methods and Monte Carlo methods are usually applied [200,201]. The accuracy of molecular docking relies on scoring functions, which are applied to determine binding affinity and ligand–target binding modes and identify the potential drug candidates [202]. Physics-based, empirical, knowledge-based, and machine learning-based scoring functions are available [202]. Additionally, new deep learning methods such as EquiBind, GNINA, DiffDock are developed to predict the binding mode between the ligand and a specific protein target [203,204,205]. Especially, Equibind and Diffdock have the potential to significantly change the VS landscape. EquiBind, an SE(3)-equivariant geometric deep learning model, can perform direct-shot prediction of the receptor binding location (blind docking) and the ligand binding pose and orientation [203]. This method significantly speeds up with better quality compared to traditional docking methods [203]. DiffDock is a diffusion generative model over the non-Euclidean manifold of ligand poses, which has fast inference times and provides confidence estimates with high selective accuracy outperforming the previous traditional docking and deep learning methods [205]. GNINA utilizes an ensemble of convolutional neural networks (CNNs) as a scoring function and improves the quality of scoring and ranking binding poses for protein-ligand complexes [204]. This method significantly outperforms SMINA/Vina in all cases including redocking, cross-docking, flexible docking, and whole protein docking tasks [204].

#### 3.1.5. MD Simulations

MD simulations have been extensively used in SBDD. In molecular docking, MD simulations can improve the flexibility of the target protein and obtain target conformations with well-defined binding cavities and flexibility for molecular docking [206,207]. Moreover, MD simulations can be applied for docking scoring and lead optimization. Combined with free energy calculations, MD simulations can accurately assess binding affinity and improve the accuracy of ranking the compounds. In lead optimization, MD simulations can be employed on the small sets of compounds (no more than a few hundred), and ligand–target interactions can be determined, which provide guidance for the further development of ligands [208]. More details about MD simulations are provided in Section 2.1 MD Simulations.

### 3.2. Ligand-Based Drug Design

In drug discovery, target structures may not be available, but some compounds against the specific target may be known. In this situation, LBDD is applied. LBDD begins with a single compound or a set of active compounds against the specific target protein. Then, compounds with physicochemical and structural properties responsible for the given biological activity are identified, which is based on structural similarities related to similarities of biological activities [5,209]. According to structure–activity relationships (SARs), the properties of the compounds are improved by designing appropriate analogs [209]. Designing can be performed in terms of structural similarity or properties. Commonly used approaches in LBDD include pharmacophore modeling and QSAR.

#### 3.2.1. Pharmacophore Modeling

Chemical features from a set of bioactive conformations of known ligands were extracted to employ the pharmacophore model. These conformations contain information about the vital interactions of ligands with the specific target [5,210]. The chemical features comprise hydrogen bond acceptors/donors, hydrophobic regions, positively/negatively charged groups, and aromatic ring regions [211]. Generation of the pharmacophore model generally includes the following steps: [212] (1) selecting a set of bioactive ligands against the specific target as the training set; (2) creating the conformation space for each ligand in the training set to characterize the conformation flexibility of the ligand; and (3) aligning the ligands in the training set and determining the chemical features to construct the pharmacophore model. Various pharmacophore model generators have been developed, such as Catalyst [213], LigandScout [214], MOE [215], PharmMapper [216], PharmaGist [217], Phase [218], Quasi [219], and UNITY [220]. Ligands with different scaffolds but similar interactions can be selected through pharmacophore-based VS. The pharmacophore model can also be combined with QSAR when aligning the ligands [221].

Classical pharmacophore models also have limitations: The models are static but are used to represent the dynamic systems. Interactions in the pharmacophore model are restricted to simple geometric features. The dynophore method [222], a combination of the pharmacophore model and MD simulations, can address these limitations. This method provides the details of ligand binding out of the traditional spherical geometry and offers statistics of different binding modes and feature frequencies during the trajectory [222].

#### 3.2.2. Quantitative Structure–Activity Relationship

QSAR, a modeling approach, unravels the relationship between bioactivities and structural properties of ligands based on the principle that bioactivities are related to structural properties [223]. Bioactivities refer to pharmacokinetic properties, including ADMET and other properties. Structural properties refer to the physicochemical properties of ligands. QSAR can rank numerous compounds according to their bioactivities, and therefore, it is extensively used in lead discovery and optimization during drug discovery. The statistical model is used for predicting the bioactivity of new ligands [223]. A reliable QSAR should meet the following requirements: [5,224] (1) obtaining the dataset of sufficient ligands (≥20 compounds) with bioactivities from the conventional experimental protocol; (2) selecting appropriate compounds to construct the training set and testing set; (3) no autocorrelation among the descriptors of the ligands (describing the chemical features of the molecule in a numerical form) that induces overpredicting or overfitting; and (4) validating the final QSAR model through internal/external validation to assure model reliability. 

Based on the method of deriving descriptors, dimension-based QSAR methods are classified as [188] (1) 1D-QSAR, relating bioactivity to global molecular physiochemical properties such as logP and pKa; (2) 2D-QSAR, relating bioactivity to structural features of the ligands, such as connectivity indices, without regard to three-dimensional representations of the features; (3) 3D-QSAR, relating bioactivity to noncovalent interactions around the ligand; (4) 4D-QSAR, additionally containing the ensemble of ligand conformations on the basis of 3D-QSAR; (5) 5D-QSAR, describing different induced-fit models of 4D-QSAR; and (6) 6D-QSAR, further combining different solvation models of 5D-QSAR. Moreover, based on the techniques of constructing the relationship between bioactivities and structural properties of ligands, QSAR methods are categorized as linear and nonlinear [225]. Linear methods consist of linear regression, multiple linear regression, partial least squares, and principal component analysis/regression [225]. Nonlinear methods include artificial neural networks, k-nearest neighbors, and bayesian neural nets [225]. To meet the QSAR requirements in drug design, various QSAR-related tools were developed, such as Cloud 3D-QSAR [226], Web-4D-QSAR [227], and DPubChem [228]. Although QSAR has its advantages, some challenges exist related to QSAR application to drug design. For example, the limitation of high-quality datasets makes it difficult to construct a reliable QSAR model. Another challenge is the limitation of descriptors used for constructing QSAR models. To address this problem, new descriptors are integrated to accurately extract structural characterizations.

## 4. De Novo Drug Design by Artificial Intelligence

The above-mentioned machine learning based target identification, binding site identification, docking prediction, develop ability predictions, affinity predictions, etc., are the whole or part of the work of drug discovery by screening the desired drug from existing compound data. EquiBind was developed to predict receptor binding location and ligand’s bound pose and orientation by applying geometric deep learning [203] ; additionally, DiffDock completed the molecular docking task by a diffusion generative approach [205]. In the following sections, we focus on reviewing the machine learning based frameworks which are relevant to de novo drug design. In other words, the state-of-the-art AI-based approaches which are able to generate novel molecules with desired properties are depicted.

Drug discovery using AI is an innovative process in which candidate molecules with desired chemical properties are created [4,229,230,231]. The number of compounds that belong to the drug would be in the 10^23^–10^60^ from the chemical space aspect, which makes computation for the mining of novel compounds a challenge task [229,232,233]. Meanwhile, since a molecule binds to a particular protein pocket so that it can inhibit or activate cellular biological functions, balancing multiple structural and physicochemical parameters is crucial in drug discovery [234]. Machine learning techniques have considerably accelerated the process of drug discovery, which can handle the complex relationship between input and output variables for high-dimensional data [235,236]. Advances in deep learning models have recently resulted in a significant progress in molecule generation [234]. While machine learning models have been used for molecular property prediction, they presented a big step forward in bridging the gap between chemical entities and drug-like properties [234,237]. In particular, combining generative techniques with various statistics and probabilistic methods is the state-of-the-art approach in this task. The goal of generative modeling is consistent with the aforementioned drug design: sample novel molecules with intersection of multiple property constraints [238].

### 4.1. Overview of the Machine Learning Based de Novo Drug Design

**Figure 3:**
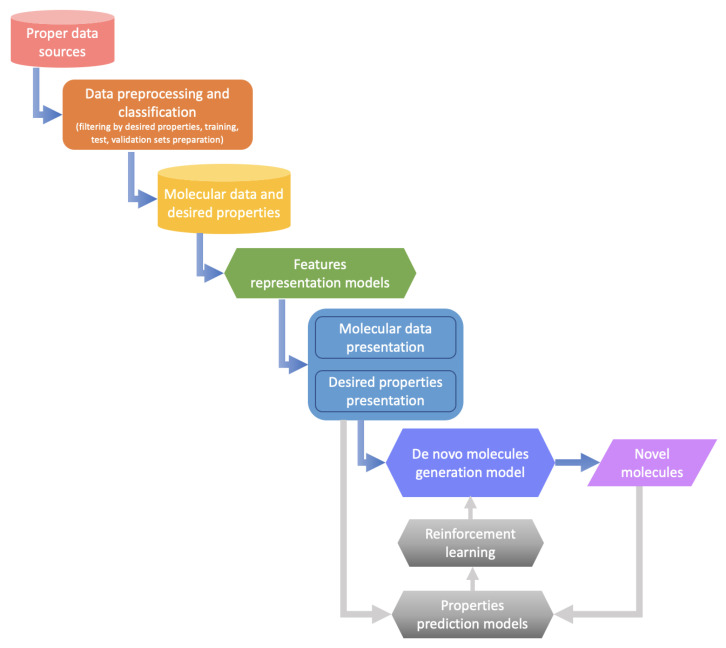
Overview of the machine learning-based de novo drug design procedure, from left-top to right-bottom: appropriate data selection; data filtering and classification; molecular data and de-sired properties storage; feature representation for molecules and properties; molecule generation by machine learning methods; generation model optimization by reinforcement learning strategy and property prediction models; de novo molecules generation.

Machine learning-based drug design involves a sequence of processes from data selection and representation to generative model construction. Figure 3 presents an overview of the machine learning-based de novo drug design procedure. In the beginning, appropriate data are selected from publicly available data sources, and property-based filtering and classification are performed to obtain molecules having desired properties for subsequent model learning. Then, sophisticated feature representation methods, such as those based on simplified molecular input line entry system (SMILES) and graphs [34,239,240], are applied to learn and represent the structures and properties of molecules. Finally, the optimal generative model is selected for de novo molecule generation based on the learned representation [229,236,238,241]. Furthermore, at the appropriate time, the generative model is optimized by combining reinforcement learning strategy and property prediction models [233,242,243].

### 4.2. Overview of de Novo Molecule Generation

The recent massive increase in AI-based de novo drug design research can be attributed primarily to the generative approach. This approach leverages deep learning strategy to learn the probability distribution of molecular data and produces continuous or discrete latent representation for molecules with property optimization. It finally maps learned probability distribution and molecule representation into novel molecules while optimizing molecular properties through the tuning of parameters of latent codes [230,231,244,245,246]. The generative approach is effective in property-based design, LBDD, and SBDD by generating both 2D and 3D molecules [239,247]. The generated novel drugs should potentially interact with therapeutic target proteins on the specific docking site, for which structural information about both proteins and molecules must be extracted during model training [248]. Hence, research efforts have been made on “structure-oriented generation” for drug design. Accordingly, “ligand-oriented generation”-based drug design focuses on structural and property information of molecules.

#### 4.2.1. Structure-Oriented Generation

Structure-oriented generation creates novel molecules that bind to specific target proteins in drug design [240]. These target proteins could be receptors, enzymes, or other structural or functional proteins, and their structural information is required for the generation task [249]. In addition to the generation process, structural information of target proteins is crucial for exploring potential receptor–ligand interactions in drug design. Besides, the generation process depends on the structures of molecules, which are usually represented by scaffolds of chemical compounds [239]. The structural information of the ligand and target protein can help explore the interaction between them [250].

In structure based de novo drug design, deep generative models are applied to learn and score docking of protein–ligand by exploring their structures and functions, and this information is used to generate de novo compound structures. The generation process applies a “fragment-based” strategy: given the initial chemical scaffold embedded in the binding site of the target protein, the pre-trained model generates molecules by sequentially adding, deleting, inserting, or replacing and linking fragments for it in an iterative manner [251,252]. In addition, with the availability of structural features of both target proteins and molecules, structure-oriented generation would allow better binding of designed drugs to target proteins [253].

In recent 5 years, the structure-based de novo drug design has applied generative machine learning models and made use of feature information such as ligand-protein complexes, protein binding sites, and bioactivity. Since the structure information of both protein and ligand are available, it is desirable to generate molecules in terms of 3D representations. A series of valuable tools for 3D molecule generation have emerged, including DeepLigBuilder, G-SchNet, RELATION, and Pocket2Mol, which employed multiple generative strategies. DeepLigBuilder applied graph generative model named ligand neural network (L-Net) to generate 3D molecules by iteratively refining existing structures, and it further combined a reinforcement learning method called Monte Carlo tree search (MCTS) to optimize the binding affinity [250]. The generative neural network (G-SchNet) learns the conditional distribution of 3D molecular structures and chemical properties [254]. RELATION also paid attention on conditional distribution by applying the variational autoencoder architecture, which consists of a 3D convolutional encoder, a LSTM-based captioning decoder and a bidirectional transfer learning module in order to transfer the features of protein-ligand complexes to latent space, for molecule generation [251]. It is not difficult to find out that the reinforcement learning and transfer learning are appropriate approaches for learning features information like binding sites, protein structure and docking scores, which were used to process molecule property optimization [234]. The Pocket2Mol has learned a probability distribution of atoms and bond types inside the pocked based on exiting atoms by adopting an auto-regression strategy, and used a graph neural network to capture features of atoms in binding pockets. For new drug sampling, this research considers the structures and geometrical constraints of protein pockets in drug design [255]. Another 3D generative model applied auto-regressive for novel molecule sampling can be found in study [240], similarly, it also used a neural network architecture to learn probability distribution of occurrences of atoms. Other studies also explored generating molecule from 1D and 2D aspects by exploiting SMILES representation of ligand and graph representation of protein binding sites, such as study [253], and they combined bioactivity affinity prediction model for generative model optimization.

#### 4.2.2. Ligand-Oriented Generation

The designed novel molecules have high binding affinity to specific proteins but low binding affinity to other proteins [233]. When compounds in the employed data are already known to bind to the target proteins, the ligand-oriented approach is used for novel structure generation [252]. Ligand-oriented approaches focus on the molecules themselves, thereby generating compounds with new chemical hypotheses while optimizing the desired properties. For instance, some approaches use the known actives of a compound for a specific target receptor for latent chemical space retrieval [256].

The properties of chemical ligands are also optimized during generation, such as ADMET, binding affinity, logP, QED, solubility, easy to synthesize, and clearance. Properties can be optimized in two ways: one is property-based generation, wherein models would learn the chemical space of molecules with desirable properties, and then, the novel molecules are generated within a desired property space [245,257]. Autoencoder is a typical artificial neural network for property-based generation, which encodes molecular data along with corresponding properties into latent space. Many de novo drug design models used the similar concept, such as CogMol applies the variational autoencoder [233], the junction tree variational autoencoder applies graph message passing network for molecular graph representation and chemical validity maintenance [230]; similarly, the graph generative model in study [238] also used message passing neural for graph generation and property control. The autoencoder in [231] then constructed a predictor to estimate chemical properties from the latent continuous representation when exploiting SMILES strings directly for encoding and decoding. A similar multilayer perceptron-based model for mapping between latent vectors and molecular properties was also trained in the popular molecule generation model named MolFlow [229]. In another method, a prediction function is applied for the desired properties of molecules and the generation model is fine-tuned in terms of a particular property by using the reinforcement learning strategy, such as constructing QSAR models, as reward functions [249,256]. GFlowNet and ReLeaSE are the well-known novel molecules generation models ap-plying reinforcement learning for property optimization [243,258]. Another common approach is to use transfer learning strategy to finetuned the model for properties such as activity optimization [259]. Whereas the influential research work, the generative model named GENTRL, not only learned a mapping from discrete molecular graphs with partially known properties to continuous latent space, but also applied reinforcement learning in the generating stage for property optimization. One of the major challenges of the ligand-based generation is the difficulty in actually synthesizing, that is, to ensure the high synthetic accessibility structures for the generated molecules [252,260]. Although some reactants and reaction rules can be used as the templates to guide the generation processes, such as RECAP [261] and SYNOPSIS [262], the structural novelty and diversity of new molecules have to be reduced.

## 5. Approaches and Techniques in Artificial Intelligence Based de Novo Drug Design

### 5.1. Datasets in AI-Based de Novo Drug Design

The first step in AI-based drug design is to learn the structures and properties of source chemical compounds to generate novel molecules that meet the requirements and expectations for desired properties such as proper binding energy, QED, and logP. Hence, appropriate molecular data are crucial for constructing the drug generative model [263]. High-throughput screening assays can act as a rich resource of bioactive molecular data, including molecular structures, chemical properties of molecular structures, molecular descriptors, side effects, clinical information, targets, and activity measurements [264]. Table 1 shows the widely used databases for AI-based drug design in recent years.

### 5.2. Descriptors/Feature Representation

Molecular descriptors/representations usually represent geometry, chemical structure, and physiochemical properties and biological activities of compounds. They are numerical vectors that are used as input for generative models [264]. Deep generative models produce molecule candidates by learning the underlying distribution of molecules on the basis of these descriptors/representation [240]. The way for representation plays a crucial role in the generative model, since efforts of deep neural networks (DNNs) have been focused on molecule representation learning. The strength of neural networks depends on their power and ability to make transformation among input, latent, and output representations [34,246].

Four categories of representation have been established: one-hot embedding-based, SMILES-based, graph-based, and 3D-based. Among these representations, the most commonly used feature representations for training DNN models are the SMILES representation and the molecular graph representation [234].

In one-hot embedding-based representation, binary vectors are used for atoms and bonds as molecular descriptors [229,249,264]. SMILES-based representation processes SMILES of a molecule directly by applying natural language processing techniques such as deep learning methods, including recurrent neural networks and long short-term memory (LSTM), which regard molecule generation as a Seq2Seq problem [34,234]. Seq2Seq is generally implemented on the basis of the encoder-decoder (encoder-decoder) framework, which is good at dealing with global information of long sequence and synthesizing contextual information of single token, to predict the corresponding alternative sequence [274,275]. The encoder transfers the SMILES strings representation into latent encodings, and the decoder transfers it into output SMILES strings by iteratively predicting probabilities of representation of particular tokens on the basis of previous information. In these methods, SMILES was firstly tokenized into a sequence of tokens, and each token was embedded into a space vector by different encoding methods such as one-hot encoding, pre-trained models [243,276]. Thus, grammar information of SMILES strings is learned for participating in the subsequent construction of generative models such as the transformer model, language model, variational autoencoder model, and generative adversarial networks [245,253]. There are recent studies used SMILES strings directly for molecule generation. The classical and representative ways are applying RNN on SMILES to generate scaffolds and corresponding attachment of molecules [276,277,278], applying self-attention mechanism model on SMILES for de novo drug design [279], and training molecule properties predictor by using SMILES [233]. Table 2 in next section shows the descriptions of the related deep learning methods.

A trend for adopting graph-based generation techniques in drug design has been increasing [233,237]. Graph-based models generate molecules regressively, sequentially predicting atoms or fragments for adding next time by learned probabilities [240]. Hence, information about atoms and bonds would be learned and represented, which correspond to nodes and edges in a graph. In graph-based representation, molecules are processed as graphs with nodes and edges corresponding to atoms and bonds by DNNs [246]. The three-dimensional information of a molecule is crucial for determining many molecular properties [236]. In the three-dimension-based representation, molecular graphs are translated into three-dimensional conformations including coordinates and distance of different connected fragments [246,254]. Details of graph-based approaches and corresponding models are discussed in the next section since most of them applying deep learning methods.

### 5.3. Deep Learning Methods for Molecule Generation

DNNs are effective and efficient in drug discovery because they have a great learning capacity and a relatively less number of parameters [241]. Deep learning models learn the distribution of molecular structures, map the structures into continuous or discrete latent vectors, and finally generate novel molecules by picking up vectors in the latent space [236,238]. The complete generation procedure applies various deep learning techniques and generative frameworks. Table 2 summarizes popular deep learning techniques used in the molecule generation step of drug design.

**Figure 4:**
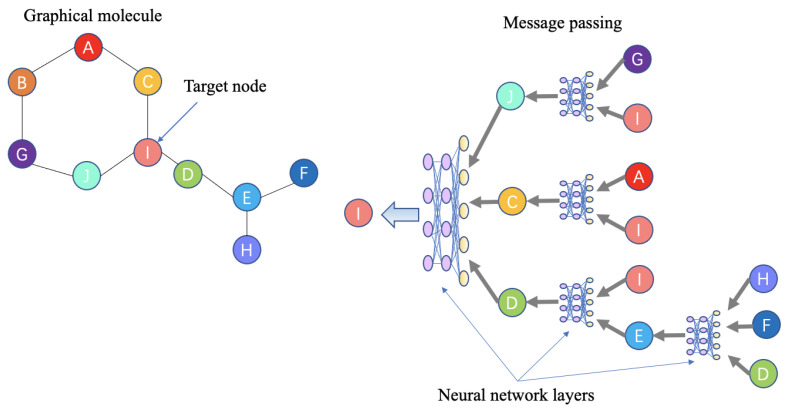
Example of a message passing neural network. The left side is an example of a graphical molecule. Ten atoms are regarded as nodes, with each node connecting with one or more nodes. The right side shows the message passing procedure for the target node I through a multilayer neural network (or one layer). The information from the directly connected nodes J, C, and D is passed to the target node I. As nodes J, C, and D have their own directly connected nodes (G, I, A, E), message is also passed by each of their neural networks. Message passing between nodes in a graph is a circular iteration process.

Notably, graph neural networks (GNNs) can learn the structural information of atoms and their neighbors and perform well during both molecule generation and property prediction. These features are attributable to the message passing networks inside GNNs. Figure 4 shows a schema explaining how this model processes and learns graphical information.

Other notable GNNs for de novel drug design include graph convolutional networks, graph attention networks, and graph generative networks [229,237,239,291].

As mentioned above, in current deep learning-based de novo drug design, models learn the probability distribution of molecular data and the mapping functions for encoding input molecular data into latent codes. They then generate new molecules by picking up vectors in the latent space based on probability distribution. This complete process can be accomplished by generative models. These models rely on machine learning-based approaches such as encoder–decoder (autoencoder), probability distribution learning, conditional distribution learning, transfer learning, and reinforcement learning [232,239,253]. The next paragraphs focus on sophisticated and classical molecule generation models.

**Figure 5:**
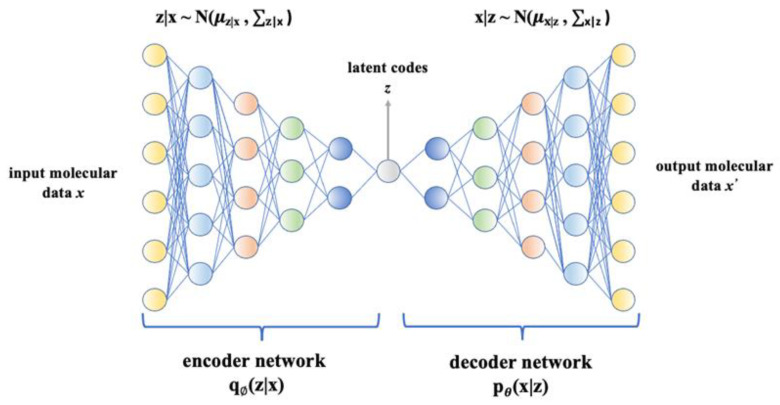
Variational autoencoder architecture. It consists of an encoder and a decoder, and they are deep neural networks. In general, the encoder maps input molecular data *x* into latent codes *z* by parameterizing a posterior distribution q_Ø_(z|x), and the decoder reconstructs molecular data from the learned distribution p_θ_(x|z).

Variational autoencoder (VAE) is a deep learning-based generative model that has been widely used in molecule design [249]. Being a probabilistic model, VAE can learn the distribution of given data and generate new meaningful data with more intra-class variations [249]. It consists of an encoder and a decoder. The encoder maps input molecular data x into latent codes z by parameterizing a posterior distribution q_Ø_(z|x), and the decoder reconstructs molecular data from the learned distribution p_θ_(x|z) [210,248,249]. Figure 5 illustrates the VAE architecture, which aims to maximize the likelihood of training data p_θ_(x), which is expressed as Formula (1) [250]:

log p_θ_(x^(i)^) = E_z_ [log p_θ_(x^(i)^|z)] − D_KL_(q_Ø_(z|x^(i)^)||p_θ_(z)) + D_KL_(q_Ø_(z|x^(i)^)||p_θ_(z| x^(i)^)) (1)

Kullback–Leibler (KL) divergence is used for measuring the difference between two probability distributions in the same space. The KL divergence D_KL_(q_Ø_(z|x^(i)^)||p_θ_(z|x^(i)^)) measures difference between posterior distribution of latent variable and its prior distribution; however in VAE, it is impossible to process the posterior distribution q_Ø_(z|x^(i)^), p_θ_(z|x^(i)^), which is introduced to approximate it [292,293]. Among Formula (1), as p_θ_(z|x^(i)^) is intractable but Kullback–Leibler (KL) divergence D_KL_(q_Ø_(z|x^(i)^)||p_θ_(z| x^(i)^)) is always larger than 0, the aim is to maximize the evidence lower bound (ELBO): E_z_ [log p_θ_(x^(i)^|z)] − D_KL_(q_Ø_(z|x^(i)^)||p_θ_(z)). As − D_KL_(q_Ø_(z|x^(i)^)||p_θ_(z)) represents the negative KL divergence between the variational approximation distribution q_Ø_(z|x^(i)^) and the distribution of the latent variable z. In order to maximize the final result, this KL divergence has to be as small as possible. This KL divergence also prevents high consistency degree between the distribution of input x and distribution of output x’.

VAE-based drug design models can exploit both SMILES strings and graphs for molecular data representation and generation [253,292,294,295]. A popular VAE strategy-based drug design is the junction tree variational autoencoder (JT-VAE). JT-VAE applies the graph message passing the network to represent the junction tree and molecular graph as latent codes. Then, it generates valid chemical substructures with the learned maximal log-likelihood to form a tree-structured scaffold, and finally assembles these substructures into the molecule [230]. Jin et al. [237] proposed a VAE-based hierarchical graph encoder–decoder that applies the message passing the neural network for graphical representation, in which each layer performs graph convolutions iteratively conditioned on the results of the last layer. Another VAE-based molecule generation model employing graph information is GENTRL. GENTRL combines variational autoencoder, reinforcement learning, and tensor decompositions. It learns mapping from discrete molecular graphs with partially known properties to the continuous latent space parameterized by distribution. Moreover, the relationships between molecular structures and their properties are encoded by the tensor decomposition method, and reinforcement learning is applied in the generating stage [242]. DeepScaffold also applies VAE for constructing a scaffold-based molecular generative model [239]. GraphVAE generates a probabilistic fully connected graph from continuous embedding and applies a graph matching algorithm to align the generated graph to ground truth [296]. For models utilizing SMILES strings, CogMol is a popular VAE-based molecule generation model that applies VAE to learn the latent space of SMILES representation along with properties such as QED. It generates novel molecules with desired properties by using a conditional latent (attribute) space sampling scheme [233]. Additionally, three-dimensional molecular structures were utilized in the VAE-based model G-SchNet that learns the conditional distribution of these structures, chemical properties, and sample molecules with target properties [254].

**Figure 6:**
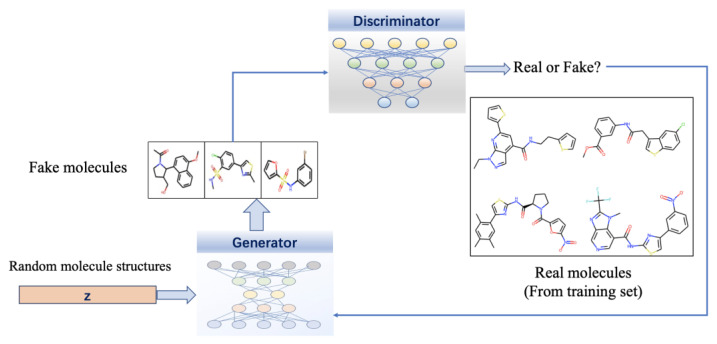
Flowchart of drug design using GANs. It contains a generator and a discriminator, and they are all deep neural networks. The generator transforms latent vectors that are sampled from a prior distribution such as Gaussian into novel molecular data samples, and the discriminator distinguishes fake molecular data generated by the generator from the actual points sampled from the distribution of training data and gives feed-back.

Generative adversarial networks (GANs) are another emerging simple but very efficient technology in drug design [297]. Compared to VEA’s traditional training method of learning through loss functions, GAN uses a more realistic comparison method to implement adversarial training, which is more interpretable. The GAN model contains two major components: a generator G, which transforms latent vectors that are sampled from a prior distribution such as Gaussian into novel molecular data samples, similar to the training samples, and a discriminator D, which distinguishes fake molecular data points generated by G from the actual points sampled from the distribution of training data with the learning boundaries between them. Hence, the generator’s task is to fool the well-trained discriminator by generating novel molecules, whereas the discriminator’s task is to improve its ability to distinguish between real and fake molecules [246]. Figure 6 presents the flowchart of drug design using GANs.

Combined with GNNs, the power of GANs can be further increased in molecule generation. One of the most widely used GAN-based drug design model is MolGAN. This model adopts GANs to directly process graphical molecular data and combines reinforcement learning to optimize specific desired chemical properties with generated molecules [298]. Since constructing sequences and graphs requires backpropagating the gradient, the training of GAN is more challenging than VEA. There are some drawbacks that are difficult to avoid for GAN: generators widely ignore random vectors leads the mappings of training data to output data are singularly deterministic; the two conflicting goals of generator and discriminator lead to a continuous drift of the learning parameters, resulting in varying degrees of distortion in the output.

**Figure 7:**
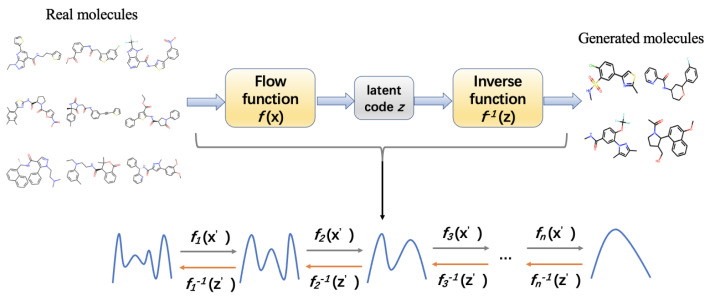
Architecture of normalizing flows. It contains a series of invertible functions for transforming molecular data into simple distribution and converting the distribution into high-dimensional molecular data for de novo drug design. Each of the functions optimizes the data distribution.

Normalizing Flow is a method proposed to overcome the shortcomings of GAN and VAE through invertible functions. Normalizing flow is a probabilistic generative model that uses simple probability distributions to simulate complex probability distributions. Composing invertible functions, the normalizing flow model learns a series of invertible transformations from the prior distribution of molecular data to simple distribution such as Gaussian. It finally converts the simple distribution into high-dimensional molecular data for de novo drug design [245,246]. Figure 7 illustrates the architecture of the normalizing flow model in molecule generation for drug design. Comparing to VAE and GAN, normalizing flow does not need any noisy data in the output, thus it allows for more robust local variance models; it is more stable during training process; and it is easier to converge. It also has its own disadvantages, namely poor interpretability, and hard to ensure the synthesizability of the generated molecules.

The normalizing flow architecture has been used in certain molecule generation models. MolFlow is a flow-based graph generative model that exploits normalizing flow to map molecular graphs and latent representations. It trains a model to generate bonds and a novel graph conditional flow to generate atoms on the basis of the bonds by leveraging graph convolution operations. Finally, the bonds and atoms are assembled in bond-valence constraints. They also train a multilayer perceptron model for mapping between latent vectors and molecular properties [229]. The hierarchical normalizing flow model, called MolGrow, generates a molecule from a single node and recursively splits every node into two; these operations are invertible and use graphical representation for node and edge attributes and feed them into an L invertible level architecture, wherein the generated latent codes fit into Gaussian distribution. Each level contains multiple blocks and linear transformations for noise separation and node merging; inside each block, three channel-wise transformations and two RealNVP layers are present [245]. GraphDF is a discrete latent variable model that applies normalizing flow for molecule generation. These discrete latent variables are sampled from multinomial distributions, and the model uses invertible modulo shift transform to sequentially map discrete latent variables to graph nodes and edges [236]. GraphNVP is a normalizing flow-based molecular graph generation model that represents the molecular graph by an adjacency tensor and a feature matrix of node attributes. It applies a continuous density model to learn probability distributions and two types of reversible affine coupling layers to transform the adjacency tensor and feature matrix into latent representations. This model first generates a graph structure and then generates node attributes [299].

In addition to normalizing flows, autoregressive model is another neural density estimator, in which a variable is predicted by the previous variable because the model decomposes the joint density as a product of conditionals [300]. The autoregressive model creates an explicit density model that can maximize the likelihood function of training data. Several autoregressive generative models are used in drug design. They build the molecular graph by refining its intermediate structure in an iterative fashion [236,301].

In addition to the above architectures, a variety of pretrained neural network models were developed for handling drug discovery related problems. The pretrained graph neural networks model 3D Infomax is an example, it can predict latent 3D and quantum in-formation by utilizing the 2D molecular graphical data, which is beneficial the down-stream tasks of molecule generation and molecular property prediction [302]. In recent years, some studies explored combining techniques of natural language processing for molecular data representation model training. The popular transformer architecture of language model BERT was applied to learn molecular representations, and the resulted model MolBert can be used for drug discovery related tasks [303]. Similar models that trained by transformer-based architecture on SMILES strings are SMILES-BERT, ChemBERTa, MegaMolBART [304,305,306], and so on. Another natural language processing related model, namely MolT5, was trained by a self- supervised learning framework on large amount of natural language text and molecule strings, which can be fine-tuned for molecule generation from natural language and molecule captioning [307]. Having such pre-trained molecular data representation models can largely improve the effectiveness and efficiency of de novo drug design.

### 5.4. Machine Learning Methods for Molecular Properties Optimization

In many drug design procedures, optimization of molecular properties (e.g., high drug-likeliness, synthetic accessibility, or solubility) is a critical step [308]. Various machine learning techniques can be applied to the input feature space, latent space, and output space. The probabilistic autoencoder can transform the features of molecular properties into latent variables [233]. Bayesian is the most popular method applied to the latent space to retrieve optimal latent solutions in the continuous latent space [34,233]. For property optimization on the output space, the most widely used strategy is to combine reinforcement learning with prediction machine learning models [233]. Statistical machine learning methods and deep learning methods help to build the classification or regression model that can predict molecular properties [263,309]. With the aid of prediction models, reinforcement learning maximizes the reward derived from the predicted scores of properties and biases the generative models, which allows the molecule generation model to achieve a high success rate in meeting the desired constraints [234,238,253]. Other optimization strategies have also been described in existing research. Modof, a generative model, applies message passing networks for encoding the difference between molecules before and after optimization at one disconnection site to connect changing fragments of a molecule and properties [308]. The Expectation–Maximization algorithm was employed in a hierarchical generative model to optimize molecular properties that mimic human experts [310].

For structure-oriented optimization, studies have been conducted to improve docking scores and activities of generated molecules for binding to specific targets. QSAR A is a classical model that is trained on docking scores from a chemical library [311]. EQUIBIND, a geometric and graph deep learning model, exploits graph matching networks, three-dimensional coordinates, and distance information-based graph neural networks (GNNs) for predicting the ligand–receptor complex structure [203]. Other studies have applied random forest, logistic regression, DNNs, and gradient boosting trees to predict the activity of generated molecules on the biological target by using molecular descriptors [259].

### 5.5. Evaluation

To understand the quality of generated molecules, evaluations are necessary. For different drug designs, different measurements from different aspects are used to evaluate the generated molecules. Table 3 displays crucial evaluation metrics in de novo drug design.

## 6. Conclusions and Perspectives

Computational biology approaches have been extensively used to facilitate drug design in target discovery, mechanism study, VS, and lead optimization. These approaches have a solid theoretical foundation, and most training data required by deep learning methods are generated using the computational biology methods. MD simulations, including force field-based simulations and ab initio simulations, continue to play an indispensable role in molecular mechanism studies, as well as thermodynamic and kinetic property research. In drug design, accurate calculation of binding energy or free energy change of ligand–target, as well as capturing structural and dynamical features of targets continue to rely on MD simulations. Compared with the ab initio method, molecular force field-based simulations can be extended to a larger scale but lacks accuracy. The QM/MM method compensates for this defect and is gradually applied for drug exploration. However, a large number of computational tasks makes it difficult for MD simulation to expand to larger scales, which limits the wide application of ab initio MD simulation. To address the computational cost concern, the CG method is designed and applied in many cases. Overall, the emergence and current wide applications of CADD, molecular docking, VS, and QSAR have accelerated drug design. Researchers have recently used AI methods to accelerate the traditional drug design paradigm and have made considerable progress. In molecular generation, generative models based on molecular graphs or strings such as SMILES or SELFIES have become the mainstream as they exhibited excellent performance in various molecular optimization tasks [258,316,317,318].

Although computational modeling of complex protein machines and AI methods have demonstrated their superior capability in drug design, several challenges remain to be solved in the current AI-based designing framework. Similar to many other areas in machine learning, molecular generator evaluation is governed by certain compound datasets. Indicators such as novelty, validity, and uniqueness are commonly used to measure model performance. Numerous generators have achieved an excellent score among these datasets. However, as some have suggested, it is difficult to confirm if the model actually learn the patterns in the training dataset. Moreover, most datasets are of low quality and cannot satisfy all demands in real drug discovery [319]. Ideal benchmark datasets should include diverse metrics for different tasks and consider practical applications; synthetic accessibility can be set as a general indicator. In addition to benchmarks and metrics in model evaluation, molecular representations play a vital role in molecular learning and generation. Two-dimensional graphs are the most conventional method to represent molecules, and such representations can be easily processed using GNNs. A typical drawback is that messages passing based on GNNs are unable to distinguish different configurations of molecules and some non-isomorphic graphs [320]. To capture spatial information, three-dimensional representations (point clouds, three-dimensional graphs, and three-dimensional grids) have recently gained considerable attention. Researchers must introduce additional units in generators to catch Euclidean symmetries in the three-dimensional space, including rotational, translational, and reflectional symmetries. Such generators are suitable for small molecular systems because of the increased complexity of macromolecules [321]. Besides, language models have received great attention in several challenging generative tasks as they learn complex molecular distributions. Language models can scale multi-modal distribution and generate larger molecules [322], while graph generative models are more interpretable. Integration of the interpretability of a graph model and the flexibility of a language model into a unified framework remains a challenge.

We suggest three potential future directions. (1) Similar to the representation learning models in natural language processing and computer vision, such as BERT, GPT3, and ViT, pre-trained molecular representation models have substantiated their potential in various downstream tasks [323,324]. (2) Domain knowledge contains a high degree of abstraction and summarization of natural phenomena and is essential for physics-informed neural networks. In computational biology, domain knowledge has been used to compute binding free energy and molecular potential and perform MD simulations [325,326,327]. To accurately estimate the binding affinity or binding free energy, rigorous but expensive methods, such as FEP or Linear Response Approximation, and their variants need to be used. Machine learning-based methods have made substantial progress in predicting binding affinity [325,328,329]. The combination of these differentiable modules with molecular generators is promising and may appreciably accelerate new drug development. (3) In addition to structural data of proteins and drugs, the availability of omics or clinical data can support drug discovery or repurposing. AI models can find hidden patterns and relations and offer more accurate prediction by using big data with different scales and types [330].

## Figures and Tables

**Table 1:** Databases for AI based drug design.

Compound Database	Description	Number of Compounds
ChEMBL [264,265]	Drug discovery database provides bioactive molecules with drug-like properties knowledge.	2,157,379
ZINC [178,231,232,233,254]	Database enables access to compounds for drug discovery.	750 million + 230 million (3D)
PubChem [233,264,266]	Public chemical database at the National Library of Medicine (NLM) collects chemical information from different data sources.	112 million
DrugBank [183,231,232,254,264]	Web resource contains drug-related information.	14,528
STITCH [264,267]	Database contains interaction information between different chemicals.	0.5 million
BindingDB [264,268]	Database for molecular recognition, which supports drug discovery related work.	1.1 million
SIDER [264,269]	Resource contains drug reactions information.	1430 + 55,730
DCDB [264,270]	Drug Combination Database	1363
GDB-11 [231,232,254,271]	Database collects and generates molecules with up to 11 atoms of C, N, O, and F by considering simple valency, chemical stability, and synthetic feasibility rules.	26.4 million
GDB-13 [231,232,254,272]	Database upgrading from GDB-11, it enumerates in a similar manner small organic molecules containing up to 13 atoms of C, N, O, S, and Cl.	970 million
GDB-17 [231,232,254,273]	Chemical universe database covers drugs and typical for lead compounds for molecules with up to 17 atoms of C, N, O, S, and halogens.	166 billion

**Table 2:** Deep learning techniques in molecules generation.

Deep Learning Techniques	Description	Applications
Recurrent neural networks (RNN)	Recurrent neural networks are similar to Markov chains with memory and feedback loops, each neuron in it would receive information from both actual time input and the previous neural [232,241,264]	SMILES strings representation [234,241,243]; generating novel and valid SMILES strings [34]; learn model autoregression [241]; construct encoder to convert discrete representations of molecules to multidimensional continuous representation [231]; estimate the probabilities of molecular data [259]
Long Short-Term Memory (LSTM)	LSTM is one kind of recurrent neural work with attention mechanism, which aims to solve the vanishing gradient problem for Recurrent Neural Networks (RNNs) [280,281]	encode SMILES strings [282]; builds sequence-to-sequence neural network for autoencoder [249]
Gated Recurrent Neural Network (Gated RNN)	One type of RNN with gated recurrent unit (GRU) containing forget gate and updating gate [283,284]	constructing encoder and decoder for SMILES sequence translation [253,285]; junction tree message passing [230]
Convolutional neural networks (CNN)	Convolutional neural networks contain sequential layers of convolution and pooling, and among them the convolution layers extract features by moving a window over the input tensors (arrays) and the pooling layers sub-sample the features [241,264,286]	construct graph convolutional neural networks [239]; grid-based 3D CNNs to predict protein–ligand binding affinity by constructing [287]
Multilayer perceptron networks (MPL)	Deep neural networks consist of multilayer perceptions, which are fully connected networks with activation functions [241]	chemical properties from latent codes [231]; mapping between latent vectors and molecular properties [229]
Multi-head attention networks	Contains encoder and decoder both with stacked self-attention and fully-connected layer inside, and the attention blocks in the network are all in the form of multi-head for receiving inputs of query, key, and value [288,289]	extract 3D conditional information of molecule [236] embed the active site graphs of target [253]
Message passing neural networks (MPNN)	A state-of-the-art and typical model for learning nodes and edges information in graph: a target node’s representation come from its directly connected nodes through a multilayer neural network (or one layer), and the message passing between nodes in the graph is a circular iteration process [290].	parameterize atom graph encoding [243] encode connected motifs information of molecule [237] graph message passing network to represent the junction tree and molecular graph into latent codes [193] learn molecular graph and rationale distribution [238]
Graph neural network (GNN)	Regarding atoms as nodes and bonds as edges, this network applies convoluting operations for graphs encoding [40,264]	atoms and bonds information representation [240,245] parameterized the encoder and decoder for atoms and bonds types [239] spherical message passing graph neural networks to extract 3D conditional information of molecule [236]

**Table 3:** Evaluation metrics in drug design.

Evaluation Metrics	Descriptions
LogP	The oil-water partition coefficient, also called the hydrophobic constant; the larger the LogP value, the more lipophilic the drug is; conversely, the smaller the LogP value, the more hydrophilic the drug is [233,239,243,245].
QED	Quantitative estimate of drug-likeness, and the value it is between 0 and 1 [239,240,250,309].
Synthesizability	The probability of the generated drug to be synthesized [233,240,277,309].
Binding affinity	The magnitude of the interaction force between receptor and ligand. It can be expressed by free binding energy [240,253].
Diversity	Generated molecules are similar in terms of the desired properties but with variety of forms [237,238,239,240,245].
Maximum Mean Discrepancy	Maximum Mean Discrepancy values between generated molecules and real molecules [236,239,245,250].
Docking score	To measure the probability of the mutual recognition between ligand and receptor through the matching principle [242,245,250].
Novelty	The quality for generated molecules to be different from existed molecules, new and unusual [238,312].
Validity	An inherent property of a drug, it represents the performance of drug in prevention, treatment, diagnosis of diseases and regulation of physiological functions [230,236,237,313].
Similarity	Similarity between generated molecules and real molecules, such as Tanimoto Similarity between molecular fingerprints [233,234].
Toxicity	The degree of poisonous or harmful that the drug would be [233,314,315].

## Data Availability

All data generated and analyzed during this study are included in this article.
